# Predicting Treatment Outcome in Major Depressive Disorder Using Serotonin 4 Receptor PET Brain Imaging, Functional MRI, Cognitive-, EEG-Based, and Peripheral Biomarkers: A NeuroPharm Open Label Clinical Trial Protocol

**DOI:** 10.3389/fpsyt.2020.00641

**Published:** 2020-07-23

**Authors:** Kristin Köhler-Forsberg, Anders Jorgensen, Vibeke H. Dam, Dea Siggaard Stenbæk, Patrick M. Fisher, Cheng-Teng Ip, Melanie Ganz, Henrik Enghusen Poulsen, Annamaria Giraldi, Brice Ozenne, Martin Balslev Jørgensen, Gitte Moos Knudsen, Vibe Gedsoe Frokjaer

**Affiliations:** ^1^ Neurobiology Research Unit, Copenhagen University Hospital, Rigshospitalet, Copenhagen, Denmark; ^2^ Department of Clinical Medicine, Faculty of Health and Medical Sciences, University of Copenhagen, Copenhagen, Denmark; ^3^ Department of Psychiatry, Psychiatric Centre Copenhagen, Copenhagen, Denmark; ^4^ Faculty of Health and Medical Sciences, University of Copenhagen, Denmark; ^5^ Department of Clinical Pharmacology, H. Lundbeck A/S, Valby, Denmark; ^6^ Department of Computer Science, University of Copenhagen, Copenhagen, Denmark; ^7^ Department of Pharmacology, University of Copenhagen, Copenhagen, Denmark; ^8^ Sexological Clinic, Psychiatric Center Copenhagen, Department of Clinical Medicine, University of Copenhagen, Copenhagen, Denmark; ^9^ Section of Biostatistics, Department of Public Health, University of Copenhagen, Copenhagen, Denmark

**Keywords:** major depressive disorder, biomarker, treatment response, serotonin 4 receptor, positron emission tomography, functional magnetic resonance imaging, electroencephalogram, cognition

## Abstract

**Background:**

Between 30 and 50% of patients with major depressive disorder (MDD) do not respond sufficiently to antidepressant regimens. The conventional pharmacological treatments predominantly target serotonergic brain signaling but better tools to predict treatment response and identify relevant subgroups of MDD are needed to support individualized and mechanistically targeted treatment strategies. The aim of this study is to investigate antidepressant-free patients with MDD using neuroimaging, electrophysiological, molecular, cognitive, and clinical examinations and evaluate their ability to predict clinical response to SSRI treatment as individual or combined predictors.

**Methods:**

We will include 100 untreated patients with moderate to severe depression (>17 on the Hamilton Depression Rating Scale 17) in a non-randomized open clinical trial. We will collect data from serotonin 4 receptor positron emission tomography (PET) brain scans, functional magnetic resonance imaging (fMRI), electroencephalogram (EEG), cognitive tests, psychometry, and peripheral biomarkers, before (at baseline), during, and after 12 weeks of standard antidepressant treatment. Patients will be treated with escitalopram, and in case of non-response at week 4 or intolerable side effects, offered to switch to a second line treatment with duloxetine. Our primary outcome (treatment response) is assessed using the Hamilton depression rating subscale 6-item scores at week 8, compared to baseline. In a subset of the patients (n = ~40), we will re-assess the neurobiological response (using PET, fMRI, and EEG) 8 weeks after initiated pharmacological antidepressant treatment, to map neurobiological signatures of treatment responses. Data from matched controls will either be collected or is already available from other cohorts.

**Discussion:**

The extensive investigational program with follow-up in this large cohort of participants provides a unique possibility to (a) uncover potential biomarkers for antidepressant treatment response, (b) apply the findings for future stratification of MDD, (c) advance the understanding of pathophysiological underpinnings of MDD, and (d) uncover how putative biomarkers change in response to 8 weeks of pharmacological antidepressant treatment. Our data can pave the way for a precision medicine approach for optimized treatment of MDD and also provides a resource for future research and data sharing.

**Clinical Trial Registration:**

The study was registered at clinicaltrials.gov prior to initiation (NCT02869035; 08.16.2016, URL: https://clinicaltrials.gov/ct2/results?cond=&term=NCT02869035&cntry=&state=&city=&dist=)

## Introduction

Major Depressive Disorder (MDD) is one of the most severe and common brain disorders worldwide with a huge impact on life quality and socioeconomic status (1, 2). It has been linked to serotonergic dysfunction, cognitive disturbances, brain network dysfunction, vulnerability to stress, neuro-inflammation, and gene by environment factors. Still, the understanding of the pathogenesis remains limited. Guidelines for MDD treatment selection are still predominantly based on simple clinical observations about overall MDD severity, and in the case of recurrent depressive episodes, it is also based on personal patient history of treatment responses. Conventional medical treatment is mainly based on intervention of the monoaminergic system in the brain, in particular the serotonin (5-HT) system. Selective serotonin reuptake inhibitors (SSRIs) act through blockage and subsequent downregulation of the serotonin transporter (SERT) (3), which presumably induces increased extracellular 5-HT levels. However, robust evidence for a central 5-HT hypofunction in patients with MDD *in vivo* is lacking (4). Roughly one third of patients suffering from MDD do not respond sufficiently to 5-HT acting drugs (5, 6), suggesting a diverse pathophysiology. The diagnostic criteria for MDD may cover a heterogenous collection of various biological entities and consequently, it is not surprising that a “one size fits all” treatment strategy is suboptimal (7). Currently, the time from starting to administer a potentially efficacious drug until it can be determined if the clinical response is satisfactory is, at best, 4–6 weeks. In clinical practice, the lack of convenient and accurate tools (e.g., quantitative and/or biological) to predict treatment response prolongs the delay from diagnosis to effective treatment and constitutes a major challenge for both clinicians and patients. Therefore, stratification of subtypes and a shift toward precision medicine, e.g., through identification of predictors of treatment response, so-called biomarker(s) that can help optimize treatment choice, is of paramount importance. Candidate biomarkers could be related to neurotransmission, specific neural networks or structural alterations in specific brain regions that can be detected by brain imaging modalities such as positron emission tomography (PET), functional magnetic resonance imaging (fMRI), and electroencephalogram (EEG) or altered biophysiological or cognitive functions (4, 8). It has also been suggested that rather than a single biomarker, an algorithm involving a set of biomarkers may prove useful to subgroup patients and predict their response to certain treatment strategies in MDD (9). Several biomarkers derived from prior large studies such as iSPOT-D, EMBARC, and CANBIND for prediction of drug response in MDD have been proposed (10–12). Here, we use multimodalities (PET, fMRI, and EEG, cognitive testing, psychometrics, and peripheral biomarkers) as part of a deep phenotyping and as a unique feature to our trial, we study modes of action in the brain on a neurotransmitter level. Thus, our trial contributes with novel insights as well as provide a dataset for cross-validation of other identified predictors of psychopharmacological antidepressant treatment response. In a non-randomized, longitudinal, open clinical trial, patients with moderate to severe depression will be treated with SSRI following Danish guidelines. In order to map neurobiological signatures of treatment, we will re-examine a subset of the cohort with neuroimaging and EEG after 8 weeks of SSRI treatment and assess cognitive changes after 12 weeks. This clinical trial is part of a larger research initiative, “NeuroPharm”, which addresses pertinent and basic questions regarding human brain disease mechanisms and seeks to predict brain responses to categories of neuro-modulatory interventions as well as treatment efficacy (www.np.nru.dk). We anticipate that this study will critically advance and inform future stratification strategies, further uncover pathophysiological and treatment mechanisms and, hopefully, guide future precision medicine approaches to optimize treatment strategies for patients suffering from MDD.

Imaging techniques have vastly increased our understanding of the underpinning cerebral mechanisms involved in MDD (13). Serotonergic dysfunction is considered a central mechanism in depression, and a recent review points at the 5-HT 4 receptor (5-HT_4_R) as highly implicated in MDD (14). For example, 5-HT_4_R agonism has shown rapid antidepressant -like behavioral effects in rodents (15), and experimental models suggest that cerebral 5-HT_4_R levels are sensitive to central 5-HT modulation in rodents (16, 17). Subsequent clinical studies from our group demonstrated that cerebral 5-HT levels can be indexed in an inverse manner through molecular brain imaging of the 5-HT_4_R by using the PET-ligand 11C-SB207145 *in vivo* (18). We here aim to evaluate 5-HT_4_R binding as a candidate predictor of antidepressant response to drugs targeting the 5-HT system in the hitherto largest cohort of MDD patients with PET brain imaging of serotonergic markers. We hypothesize that 1) patients with MDD differ in cerebral [^11^C]SB207145 binding at baseline compared to healthy controls; 2) [^11^C]SB207145 binding at baseline in patients with MDD predicts remission after 8 weeks of pharmacological serotonergic intervention; 3) After 8 weeks of serotonergic intervention, patients with remitter status have a significantly greater reduction in cerebral [^11^C]SB207145 binding than non-responders. For an overview of primary hypotheses for other modalities, see **Appendix 1**.

fMRI can be used to assess regional activity and resting state functional networks in MDD. One systematic review found abnormal (negative bias) reactivity in amygdala responsiveness to facial expressions and emotional stimulation in patients with MDD versus healthy controls (19), and pre-treatment low amygdala reactivity has shown to be predictive for antidepressant treatment response (20). A study with 70 patients with MDD was able to predict treatment recovery with ~80%, by investigating amygdala reactivity to facial emotions and its interaction with history of early life stress (21). Another study from our group showed that amygdala reactivity was associated with brain 5-HT_4_R binding and hence putatively extra synaptic 5-HT levels in healthy individuals. This established a plausible connection between 5-HT levels and amygdala activation, both involved in emotional cognitive processes (22). This exemplifies how a multimodal PET and fMRI strategy can highlight molecular mechanisms mediating drug effects on brain function (23). Resting state fMRI (rs-fMRI) measures fluctuations in fMRI signal during the absence of an explicit task and is widely used to assess distributed intrinsic networks such as the “default mode network” (24). Alterations in rs-fMRI connectivity have been described in MDD (25) and a recent study suggested that rs-fMRI can define subtypes of MDD and predict antidepressant treatment response (26), but this has been contested by others (27). Although promising, brain imaging studies have in general been inconclusive and with small sample sizes (9, 28). In the current trial, we will use task-based and rs-fMRI in a large cohort of patients with MDD and investigate the association between 5-HT_4_R levels (as a proxy for brain serotonin levels) and the clinical outcome of SSRI treatment.

EEG, a monitoring technique for direct ongoing neural activity, has been reported to be associated with treatment response in MDD [see, e.g., review (29)]. Prior studies have found that treatment responders have higher cortical alpha activity (30) and higher theta activity at rostral anterior cingulate cortex compared to treatment non-responders (31, 32). Of note, these biomarkers were derived from the resting EEG data, which is relatively easy to implement in the clinic. Furthermore, earlier evidence from event-related-potential (ERP) studies have suggested that ERP biomarkers such as auditory P300 (a positive waveform around 300 ms after stimulus onset) and loudness-dependence of auditory evoked potentials (LDAEP) can be predict drug treatment response (33, 34), and are linked to the serotonergic transmitter system (35). In the current trial, the predictive values of pretreatment EEG/ERP biomarkers will be examined.

Disturbances in cognitive processes including memory, attention, and executive functions are commonly reported in MDD (36) and contribute to psychosocial impairment and workforce disability (37). In addition, affective bias in information processing (i.e., favoring negative information over positive information at different levels of information processing) has been proposed as a central mechanism in the development and maintenance of depressive symptoms (38) which is also predictive of later treatment response to antidepressant drugs (39). Notably, cognitive disturbances do not always resolve with the remission of a depressive episode, suggesting a dissociation between core mood and cognitive symptoms in MDD (40). Combined with the low cost and relative ease of testing in a clinical setting, this distinguishes cognitive disturbances as a promising marker for stratification of depression subtypes as well as an important target for antidepressant treatment. In the present study, we therefore aim to map a broad range of cognitive disturbances in MDD, including both cold (non-emotional) and hot (emotional) cognitive processes, and explore whether they may be used to characterize clinically relevant subgroups in MDD. Based on earlier observations in healthy individuals, we expect memory performance to map onto hippocampal 5-HT_4_R availability (41) and possibly affective bias in verbal memory in MDD (42).

Evidence of inflammation-associated MDD has emerged over the years (43). Patients with MDD show elevated levels of inflammatory markers in peripheral blood (44) which may affect treatment response such that higher levels are associated with worse response (45). It has also been suggested that patients with MDD have higher levels of activated microglia, as illuminated with PET (46). Proinflammatory cytokines may influence the 5-HT homeostasis in the brain by acutely upregulate SERT through intercellular pathways (i.e., linked to p38 mitogen-activated protein kinase) and presumably thereby reduce synaptic 5-HT levels (47). Interestingly, cognitive dysfunction, a prevalent symptom in depression, also appear to be linked to an inflammatory response (48). We here aim to determine if higher levels of systemic inflammatory markers are associated with 5-HT_4_R brain binding, depression status at baseline and clinical treatment response.

Another area of interest is the association between MDD and signatures of early aging. There is an increased mortality and prevalence of age-related diseases in recurrent depression (49, 50). Oxidative stress on nucleic acids is a general element of aging and has been suggested to be an underlying biological mechanism of the accelerated aging observed in depression (51). Previous research from our group has found evidence for such a link, both in studies of psychological/biological stress and oxidative stress in patients and in rodent models of depression (52–54). Earlier findings indicate alterations in levels of oxidative stress during antidepressant treatment and it is hypothesized that treatment response is related to a transient increase in oxidative stress levels, perhaps due to neurotrophic processes and/or peripheral changes in energy metabolism (55–57). Urinary 8-oxodG and 8-oxoGuo are sensitive and specific markers for systemic DNA/RNA damage from oxidation (58). We here aim to investigate urinary 8-oxodG and 8-oxoGuo as a predictive biomarker for antidepressant treatment response, its association with changes in psychopathology, structural and functional brain changes, and markers of psychological and biological stress. Additionally, we will investigate whether hormonal [estradiol, testosterone, progesterone and follicle-stimulating hormone (in females)] and metabolic status can predict antidepressant treatment response and explore whether these associations are related to genetic make-up (specified below), psychopathology and the occurrence of early life stress using self-reported childhood adverse events and parental bonding quality questionnaires, which also may interact with the 5-HT system (59, 60).

Sexual dysfunction (e.g., low sexual desire, arousal difficulties, and anorgasmia) is a prominent feature of MDD, which often leads to a decline in quality of life (61, 62). Lack of interest to what is usually pleasurable, i.e., anhedonia, is a core symptom in MDD and may also be reflected in reduced sexual desire/interest. Sexual dysfunction, in particular anorgasmia and sexual arousal difficulties may further be linked to serotonergic dysfunctions (63) as seen in MDD. As a complicating factor, impaired sexual health related to MDD may worsen with antidepressant treatments targeting the 5-HT system. For example, in a group of 704 patients with MDD treated with an antidepressant drug (SSRI) or serotonin-norepinephrine reuptake inhibitor, about half of them developed or experienced worsening in their decreased sexual desire as a side-effect, which was also associated with reduced quality of life, lower self-esteem and adverse effects on mood and partner relations (62). We currently do not know which patient characteristics predict sexual dysfunction in response to SSRI treatment. However, differences in individual serotonergic brain architecture and/or serotonergic response to antidepressant treatment (e.g., SSRI) may play a role. In this study, we aim to map the frequency and predictors of SSRI induced sexual dysfunction and determine if serotonergic tonus (measured by 5-HT_4_R PET binding) pre-treatment, or changes in response to SRRI treatment, is associated with sexual desire and/or development of SSRI-induced sexual dysfunction in MDD.

Previous findings from our group have repeatedly demonstrated a coupling between key features of the 5-HT system and hypothalamus- pituitary- adrenal axis (HPA-axis), which regulates the release of the stress-hormone cortisol (64). Such HPA-axis dynamics can be measured by the cortisol awakening response. Our results support that both serotonin transporter availability (65), and serotonergic tone or direct capacity for 5-HT_4_R agonism (64) support a healthy cortisol response to HPA-axis stimuli. A well-functioning and dynamic HPA-axis is critical for coping with everyday life stressors, and HPA-axis dysregulation is a prominent feature of MDD. Although heightened CAR is associated with relapse of depressive episodes in patients with a history of depression (66), in the more advanced depressed stages, i.e., chronic depression, HPA-axis dynamics are blunted as opposed to recent-onset depression (67). Notably, normalization of the HPA-axis in response to SSRI treatment appears to protect against relapse (68). Thus, the SSRI treatment response is likely to depend on restoring HPA-axis dynamics at least in a subgroup of MDD patients. In this trial, we will assess CAR in patients with MDD, the effect of SSRIs on CAR, investigate its association with baseline 5-HT_4_R distribution, as well as evaluate CAR as a predictor of antidepressant treatment outcome.

## Materials and Methods

**Figure 1:**
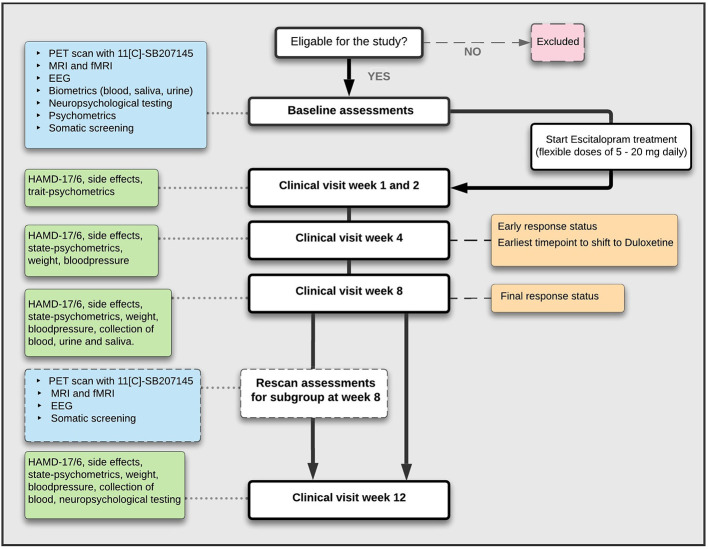
Flowchart of study trial assessments for patients with MDD.

**Figure 1** shows a flowchart of the scheduled data collection over the 12 weeks of pharmacological drug treatment of patients with MDD. Healthy controls (HC) will be recruited as specified below. Patients will be examined before (at baseline; week 0) and after 1, 2, 4, 8 and 12 weeks of SSRI treatment has been initiated. Depression-severity will be monitored by the Hamilton Depression Rating scale 17 items (HAMD_17_) and its subscale of 6 items (HAMD_6_) (69). A subset of patients will be offered re-examination with PET, fMRI, and EEG after 8 weeks of treatment, to assess changes from baseline and its association to treatment response. Patients from the whole spectrum of treatment responses (from poor to excellent) will be invited in a continuous fashion for this part of the study until allotted re-examinations are completed. All patients will also repeat cognitive testing at week 12. The power analysis in preparation of the study was primarily anchored to the PET modality. We estimated that we needed to include 100 patients to reach a statistical power of 80% to detect an association between treatment response (binary classification, i.e., remitters vs non-responders, see response-definition below) and baseline 5-HT_4_R non-displaceable binding potential (BP_ND_). These calculations were based on an expected 20% maximum drop-out, ~50% remission rate after 8 weeks of treatment (5, 6) and an expected difference of 8% in 5-HT_4_R binding between remitters and non-responders, corresponding to the previously found effect sizes on 5-HT_4_R change in BP_ND_ after fluoxetine treatment (18). Calculations were further based on an average BP_ND_ of 0.71 and a standard deviation of 0.073 (18, 70). With a rescan subgroup of approximately 40 patients, and a Gaussian distribution of change in BP_ND_ with an SD of 0.08 (log scale), we had an expected power of 80% to identify a significant association between longitudinal changes in BP_ND_ and changes in HAMD_6_ (i.e., secondary clinical outcome, continuous scale).

### Participants

**Figure 2:**
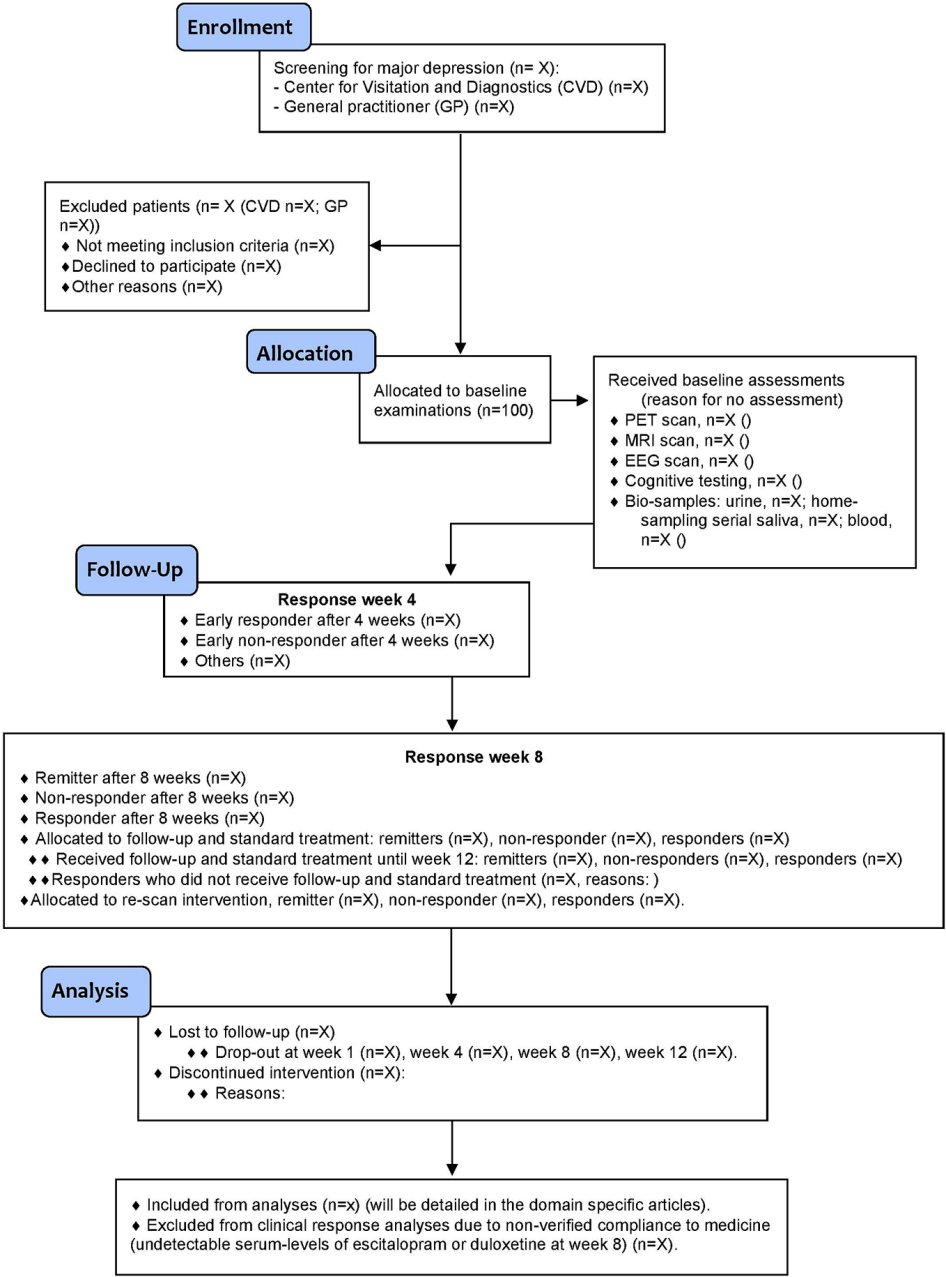
Flow diagram (CONSORT) of the NeuroPharm trial.

Patients are recruited from a central referral center within the mental health services in the Capital region of Denmark or directly referred from one of five general practitioners in collaborations with the study group (see **Figure 2** (CONSORT) for details). Data from healthy controls for the purpose of baseline comparisons to patients with MDD are available from a pre-existing database on site (71). The healthy control reference population will be supplemented with newly recruited healthy controls from a local volunteer database (www.nru.dk), as necessary.

#### Inclusion Criteria for Patients

Patients between 18 and 65 years of age with a moderate to severe, single, or recurrent episode of MDD consistent with the Diagnostic and Statistical Manual of Mental Disorders -5 (DSM-5) and International Statistical Classification of Diseases and Related Health Problems -10 (ICD-10) criteria will be recruited by a trained clinician. Inclusion requires a total score of >17 on HAMD_17_ at baseline and the diagnose is confirmed by using the diagnostic tool Mini International Neuropsychiatric Interview (72). In addition, all patients are diagnostically verified by a specialist in psychiatry before final inclusion.

#### Exclusion Criteria for Patients

Patients with a duration of their present depressive episode exceeding two years are not included. No more than one antidepressant treatment attempt in the current episode prior to inclusion is allowed and only patients with no antidepressant medication within the last two months are eligible. Patients with known contraindications or previous non-response to an SSRI drug after an adequate trial as well as a prior or present history of other primary axis I psychiatric disorders are not included, i.e., MDD must be the primary diagnosis. Other exclusion criteria are: severe somatic illness; substance or alcohol use disorder; insufficient language skills to undergo clinical assessments; acute suicidal ideation or psychosis; patients who are deemed by a psychiatrist to require other forms of antidepressant treatments; pregnancy or breast feeding; use of any CNS drug that cannot be washed out prior to participation (e.g., metoclopramide, ondansetron, serotonergic migraine medicine, clonidine); medical conditions interfering with measurements, contraindications for PET and/or MRI scans; exposure to radioactivity >10 mSv within the last year; severe sensory or intellectual impediments interfering with comprehension of procedures or assessments and lastly any history of brain injury (i.e., loss of consciousness and amnesia or symptoms of concussion disorder).

#### Inclusion and Exclusion Criteria for Healthy Controls

Enrolled HC will be sought to match the patient population by gender and age distribution. All HC will be screened for MDD using a self-reporting questionnaire (major depression inventory) (73). The HC meet the same inclusion and exclusion criteria as required for patients apart from psychiatry related issues (e.g., no current or history of mental illness or unstable somatic condition).

### Treatment and Investigation Program

#### Baseline Assessments Before Treatment

Each patient will receive a basic physical screening including somatic status, routine blood samples, electrocardiogram including QTc interval and collection of toxicology urine tests [The Rapid ResponseTM Multi-Drug Test Panel (Urine)] for detection of drug abuse within the last month. Women are screened for pregnancy through self-reported use of contraceptives and a pregnancy urinary test if relevant. All study-participants will undergo baseline assessments of brain imaging with 11C-SB207145 PET and fMRI; EEG-examination; cognitive testing, collection of questionnaires and biological material (venous blood, urine, and saliva) as specified below. All HC will receive corresponding baseline assessments.

##### Clinical Procedure After Treatment Initiation

After completion of baseline examinations, patients will receive flexible doses of the SSRI drug escitalopram, initially 5 mg for 3–5 days depending on side effects (e.g., nausea), followed by 10 mg daily until their first follow-up visit and further adjusted individually to a maximum dose of 20 mg. Escitalopram was chosen as it binds with high selectivity to the 5-HT transporter and has minimal affinity for other receptors (74). Patients are allowed short-time treatment with cyclopyrrolone (a nonbenzodiazepine hypnotic agent) or oxazepam (a benzodiazepine) to reduce anxiety and sleep disturbances which may be prominent in the initial treatment phase and have shown not to influence treatment continuation (75), but all are requested to avoid use 3 days prior to brain scans. Clinical follow-up sessions with a study physician or trained research assistant are scheduled in an out-patient clinical setting after 1, 2, 4, 8 and 12 weeks of treatment to evaluate treatment response and side effects. Visits can deviate a maximum of one week from the original time scheduled. No cognitive behavioral therapy or other psychotherapy program is provided during clinical visits. No treatment (pharmacological or psychotherapeutically) other than the medical monotherapy provided in this study is allowed elsewhere during the trial. At week 4, early non-responders (see definition below) or patients with unacceptable side effects are offered to switch to a standard second line antidepressant treatment; duloxetine (individually adjusted doses of 30–120 mg per day), which is a serotonin-norepinephrine reuptake inhibitor. Duloxetine was chosen according to clinical guidelines for second line antidepressant treatments. Cerebral 5-HT_4_R binding in humans is unaltered by injection of escitalopram (76). No prior *in vivo* studies have investigated the effect of duloxetine on 5-HT_4_R binding in the human brain, but *in vitro* work has shown that duloxetine has negligible affinity for the 5-HT_4_R (77). That is, none of the pharmacological compounds directly target 5-HT_4_R’s. The week 4 timepoint is in line with national guidelines in Denmark for switching to a second-line antidepressant treatment (4–6 weeks). Since our cohort receives frequent clinical follow-up sessions, patients can reach max dose of escitalopram (20 mg daily) already after 2 weeks. As such, switching after 4 weeks is considered appropriate for early non-responders in this trial set-up. All antidepressant medicine will be provided for free to improve compliance. Compliance will be assessed by serum escitalopram/duloxetine blood tests after eight weeks of treatment as well as tablet count at each follow-up. At each visit, depressive symptoms are rated using the HAMD_17_ and the HAMD_6_ subscale. HAMD_6_ captures core symptoms of depression more directly (and disregards sleeping quality), and has been found to be sensitive to antidepressant treatment response (69). Potential side effects due to intervention will be monitored at each visit using the “Udvalg for Kliniske Undersøgelser” scale (78). To ensure agreement and allow alignment of ratings, HAMD_17/_HAMD_6_ co-ratings between all the clinical investigators will be performed regularly during data collection. A maximum of 20% deviation from the “gold-standard” chief psychiatrist is allowed, or else a new satisfactory co-rating is needed before independent rating of study participants.

##### Clinical Response Status

###### Primary Clinical Outcome Measure

**Figure 3:**
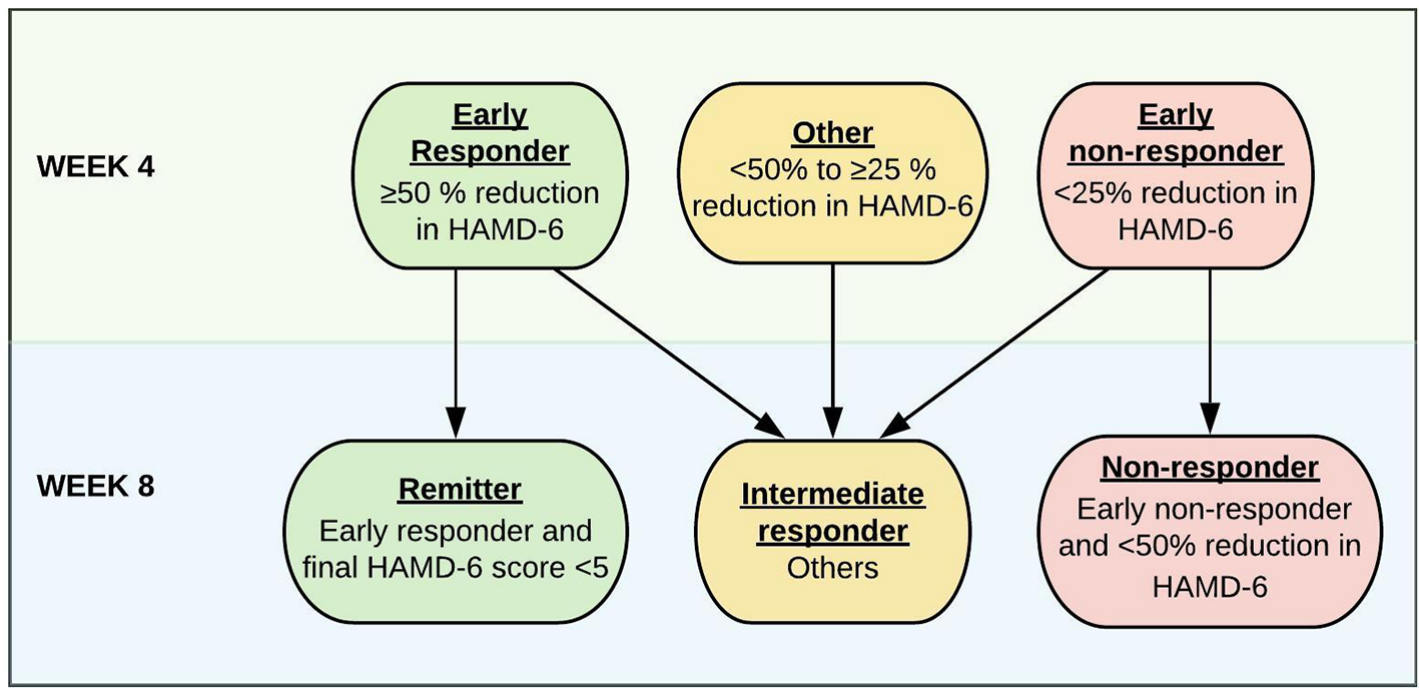
Response categorization for patients with MDD after 4 and 8 weeks of antidepressant treatment based on changes in HAMD_6_ score.

The primary outcome measure is categorical and built to capture patients with an early as well as sustained, either excellent or poor response to treatment. Patients are classified as either “remitters”, “non-responders”, or “intermediate responders” after 8 weeks of treatment. These categories are based on percentage changes of depressive symptoms from baseline, as measured by HAMD_6_. Remitters must have ≥50% reduction in HAMD_6_ at 4 weeks (early responders) and a HAMD_6_ score <5 after 8 weeks of treatment. Non-responders have <25% reduction in HAMD_6_ after 4 weeks (early non-responder) and <50% reduction in HAMD_6_ after 8 weeks of treatment. Patients who do not meet the criteria above are defined as “intermediate responders” at week 8. The primary predictor analyses are directed to predict treatment response in a binary fashion (either remitter or non-responder (see **Figure 3**).

###### Secondary Clinical Outcome Measure

As a secondary outcome, we use a continuous response measure, i.e., HAMD_6_ changes from baseline at week 8 divided by HAMD_6_ at baseline, to allow analyses of the association between antidepressant treatment response and baseline characteristics or treatment-induced changes in the neurobiological modalities of interest.

### Examination Modalities

#### PET Imaging and Quantification of 5-HT_4_R Brain Binding

PET scans are conducted using a high-resolution research tomography Siemens PET scanner (CTI/Siemens, Knoxville, TN, USA) (256 × 256 × 207 voxels; 1.22 × 1.22 × 1.22 mm). Participants are positioned uniformly in spine position and a specialized head holder is applied to reduce head motion during the scan. All participants undergo a 6 min transmission scan and are given an intravenous bolus of approximately 600 MBq of the PET tracer ligand [^11^C]SB207145. The bolus is administered over 20 s followed by a 120-min dynamic PET data acquisition. The radioligand is synthesized immediately prior to injection as described elsewhere (79).

#### Preprocessing and PET Quantification

The 120 min dynamic PET acquisitions are reconstructed into 38 time frames (6 × 5 s, 10 × 15 s, 4 × 30 s, 5 × 2 min, 5 × 5 min, and 8 × 10 min) using a 3D-OSEM PSF algorithm (16 subsets and 10 iterations) (80) and TXTV-based attenuation correction (81). For motion correction, the AIR 5.2.5 software will be used (82), aligning each PET frame to the first 5-min frame. Structural 3-Tesla MRI scans will be used for co-registration of the PET images with SPM8 software. Automatic delineation will be carried out in a user-independent manner in PVElab software (83) and mean tissue time activity curves for grey matter volumes will be extracted for kinetic modeling. No partial volume correction will be performed because of the high resolution of the scanner. Regions of interest (ROI) have been chosen due to their known relevance in mood disorders and abundance of 5-HT_4_R density (84). The selected ROIs for the primary analyses are neocortex, putamen, caudate nucleus and hippocampus. Co-registration and correct ROI placement for all subjects will be inspected in three planes by a trained investigator. PMOD version 3.0 (PMOD, Zurich, Switzerland) will be used for kinetic modeling and quantification of the 5-HT_4_R binding is performed using non-displaceable binding potential (BP_ND_) as the final outcome measure. The simplified reference tissue model will be used with cerebellum (excluding vermis) as reference region which previously has been validated in humans (76). BP_ND_ is defined as:

$$BP_{ND}=\frac{f_{ND}\times \text{Bavail}}{KD}$$

where f_ND_ is the tissue free fraction of non-protein bound ^11^C-SB207145, Bavail is the concentration of available 5-HT_4_R and K_D_ is the dissociation constant for the tracer at equilibrium. Thus, BP_ND_ is proportional to the density of 5-HT_4_R.

#### MRI and fMRI Imaging

All participants are screened for MR-compatibility and thoroughly instructed how to perform the fMRI paradigms by a trained study assistant who uses standardized instructions. All MRI scans for patients will be acquired using the same Siemens 3-Tesla Prisma scanner with a 64-channel head coil. High-resolution structural T1- and T2-weighted MR images will be acquired. Blood oxygenation level dependent fMRI scans will be obtained during a commonly used emotional faces paradigm (85, 86), reward-related guessing paradigm (87, 88) and a 10-min rs-fMRI scan. During the rs-fMRI scan, participants are asked to close their eyes, let their mind wander and to not fall asleep. All structural scans of patients will be screened for pathological abnormalities by a medical specialist in radiology.

#### EEG

EEG data is recorded using a 256-channel HydroCel Sensor Net system (EGI, Inc., Eugene, OR) at 1,000 Hz with 0.1–100 Hz analog filtering where vertex electrode serve as the reference. Impedances across all electrodes are kept below 50 kΩ. EEG/ERP recording at baseline included: resting EEG (with eyes closed and open), two-tone auditory oddball and the LDAEP tasks. The same EEG/ERP recording will be re-tested in a subgroup of patients after 8 weeks of treatment.

##### Resting EEG

Resting EEG is recorded during four 3-min periods with a counterbalanced order of OCOC (O for eyes open, C for eyes closed) or COCO between subjects. Participants are instructed to remain still and relax, avoid eye-blinks and movements and to relax chin muscles during recording. Absolute and relative powers are computed using the following frequency bands: δ (1–4 Hz), θ (4–8 Hz), α (8–12 Hz), and beta (8–30 Hz). In addition, alpha peak frequency (APF) is identified by the frequency at maximal absolute power from the spectral range of 7–13 Hz. Frontal alpha asymmetry will be calculated using alpha power with the formula of (F4 – F3)/(F4 + F3) (Arns et al., 2015). Furthermore, theta activity will be extracted from anterior cingulate cortex with exact low-resolution electromagnetic tomography (eLoreta).

##### Task Elicited ERPs

The two-tone auditory oddball paradigm consists of two acoustic stimuli with different frequencies. Participants are presented with a series of standard tones (500 Hz) and deviant tones (1,000 Hz) binaurally through inserted earphones (Etymotic Research Inc., ER 3C). They are instructed to press a button when the deviant tones are presented while ignoring the standard tones. ERP components such as N1 and P3 will be computed, both peak latency and amplitude (baseline to peak) will be extracted by the averaged trials. Participants are presented with five acoustic stimuli with different intensities (60, 70, 80, 90, and 100 dB SPL) in the same frequency of 1,000 Hz. No response is needed. The primary outcome is the slopes of peak-to-peak N1/P2 amplitudes extracted from the average trials at each intensity. A more comprehensive description of the EEG data will be presented in the subsequent reports.

#### Cognitive Testing

All participants undergo cognitive testing using selected tasks from the novel test battery EMOTICOM, assessing affective and social cognition including emotional face recognition, emotional threshold detection, theory of mind, and moral emotions (89). In addition, affective memory (90), working memory, reaction time and IQ will also be assessed. Testing is planned and conducted by trained neuropsychologists prior to start of drug intervention and again after 12 weeks of treatment.

#### Psychometrics

Apart from clinical visits including HAMD_17/6_ ratings, patients will apply self-monitoring during the study period and fill out Danish versions of online questionnaires throughout the study. All questionnaires will be imported directly to an internal database through LimeSurvey, a free and open source software. Before EEG scans and cognitive testing, all participants will report their current mood state using an in-house Likert-scale. An adjusted Likert-scale will be filled out after each MR-scan. During visits at week 4, week 8, and week 12, patients are also asked to fill out a comprehensive set of self-rating state questionnaires (see **Table 1** for a full overview). Healthy controls will be asked to fill out selected state questionnaires as part of their baseline assessments.

**Table 1:** Table over questionnaires obtained throughout the study.

Questionnaires	Time point					
	Baseline	Week1	Week 2	Week 4	Week 8	Week 12
**MINI**	X					
**HAMD-17/6**	X	X	X	X	X	X
**UKU**		X	X	X	X	X
**NEO-PIR**	X					
**CATS**	X					
**EHI**	X					
**OS-FHAM**	X					
**PBI-mother/father**	X					
POMS*	X				X	X
Likert-scale*	X				X	X
**BDI-II**	X			X	X	X
**MDI**	X			X	X	X
**PSS**	X			X	X	X
**SHAPS**	X			X	X	X
**RRS**	X			X	X	X
**CSFQ_F_C**	X			X	X	X
**SUSY item 32**	X			X	X	X
**Activity**	X			X	X	X
**GAD-10**	X			X	X	X

Trait questionnaires at baseline includes personality traits with NEO-PIR (91); Child Abuse and Trauma Scale (CATS) (92) a survey about early life stress which has shown to be able to modulate the serotonin system in the brain (93); handedness with Edinburgh Handedness Inventory (EHI) (94); an in-house version of the Family History Assessment module (FHAM) questionnaire, i.e., “Online Stimulant” (OS)-FHAM; Parental Bonding Inventory (PBI) (both mother and father) (95). State conditions included a self-rating questionnaire of Profile of Mood States (POMS) (96); an in-house Likert-scale; Beck’s Depression Inventory-II (BDI-II) (97); Major Depression inventory (MDI) (98); Cohen’s Perceived Stress Scale (PSS) (99, 100); Snaith-Hamilton Pleasure Scale (SHAPS) (101); Rumination Response Scale (RSS) 102; Changes in Sexual Functioning Questionnaire (CSFQ) (103); “Sundhed og Sygelighed” Sex Quality Questionnaire item 32 (SUSY-item 32) (104); an in-house questionnaire about daily physical activity (71); and Generalized Anxiety Disorder-10 (GAD-10) (105). * Collected in immediate extension to EEG and MR examinations or cognitive testing.

#### Biomaterials

##### Blood

At baseline, all participants will be screened for basic somatic status to exclude somatic conditions with possible influence on depressive symptoms. Blood samples will be collected throughout the study (see **Table 2** for a full overview) for determination of inflammatory status (high sensitivity C-reactive protein, tumor necrosis factor-α, Interleukin-6, -18, and -10) (106–109); epigenetic variations (SERT, FKBP Prolyl Isomerase 5, Catechol-O-Methyltransferase (COMT), monoamine oxidase-A, glucocorticoid-, estrogen-, oxytocin receptor and oxytocin gene-methylation); extraction of DNA for genotypes of relevance (rs41271330 (110), serotonin-transporter-linked polymorphic region (5-HTTLPR) (70), COMT, Brain-Derived Neurotrophic Factor val66met) and ABCB1, FZD7, and WNT2B (that presumably influence responsiveness to pharmacological antidepressant treatment (111)). At week 8, serum samples of the antidepressant drug (i.e., escitalopram or duloxetine) are collected as trough concentrations in steady state, with primary purpose of monitoring compliance. The samples will be stored at −20°C (or −80°C for plasma EDTA samples) until analyzation in batches at completion of the trial. Quantification of escitalopram and duloxetine in serum will be performed at the Laboratory of the Danish Epilepsy Centre, Filadelfia, using a routine UPLC-MS/MS method developed in-house. Standard operating procedure instructions have been established before trial initiation and will be followed during the assessment of all biomaterial.

**Table 2:** Somatic status and biomaterial assessed at various timepoints throughout the study.

Analysis	Sample	Timepoint		
		Baseline	Week 8	Week 12
Somatic blood-sample screening	Hemoglobin, white blood cell count, metamyelo.+myelo.+promyelocytes. C-reactive protein.	X	X	X
	Na+, K+, Creatinine	X		
	ASAT, ALAT, GGT, LDH, BAP	X		
	Albumin, Coagulation factors II+VI+X, thrombocytes	X		
	B12, Folate	X		
	25-OH-vitamin D	X		
	Blood sugar, HbA1c	X		
	Triglycerides, total-cholesterol, HDL, LDL	X		
	TSH, Ionized Calcium	X		
	Estradiol, testosterone, progesterone, FSH (females)	X		
Somatic examination	Electro Cardiogram (ECG) Neurological status Somatic status	X X X		
Compliance to medicine control	S -escitalopram or S -duloxetine		X	
Biobank	Inflammation and cytokines (hsCRP, TNF-α, IL-6, IL-18 and IL-10)	X	X	X
Biobank	Epigenetics (5-HTT, glucocortocoid-, FKBP5, COMT, MAO-A, estrogen-, oxytocin receptor and oxytocin gene-methylation)	X	X	X
Biobank	Genotypes (rs41271330, 5-HTTLPR, COMT, BDNFval66met)	X		
Biobank	Gene transcription profiles (mRNA and microRNA, ABCB1, FZD7 and WNT2B)	X	X	X
Oxidative stress	Urine (8-oxo-dG and 8-oxo-Guo)	X	X	
Biobank	Saliva (Cortisol awakening response)	X	X	

##### Saliva

Saliva will be collected to determine the total cortisol output across one day as well as dynamics of the HPA-axis, as indexed by CAR. Serial saliva samples will be sampled at home and collected at baseline and at week 8 (see **Table 2**). Those visits will be placed as close to the PET-scan day or week 8 visit as possible, and patients are instructed to take samples immediately after awakening and again after 15, 30, 45, and 60 min, at 12, 6, and 11 pm. Participants are also instructed to collect saliva samples preferably during weekdays, not perform strenuous exercise <2 h and not to have any oral intake or brush their teeth <1 h prior to sampling. Cohen’s Perceived Stress Scale and basic information about sleep and food intake will be filled out in conjunction with the home-sampling. All participants receive careful training in saliva collection, instructions of home-sampling procedures; cold storage of samples and fast delivery either by mail or personal delivery to the laboratory facility for preparation. When received, salivary test-tubes are centrifuged and stored at −80°C until later single-batch analysis.

##### Urine

Spot-urine samples will be collected at baseline and week 8 visits for patients (see **Table 2**) in 2-ml Eppendorf tubes and will be stored at −20°C for later single-batch analysis. Apart from pregnancy and drug-screening (see *Baseline Assessments Before Treatment*), all urine samples will be analyzed for 8-oxodG and 8-oxoGuo markers for systemic DNA/RNA damage with ultra-performance liquid chromatography with tandem mass spectrometry and normalized to urinary creatinine (112).

### Statistical Analyses

#### Evaluating Associations Between Baseline Measures, Changes From Baseline Measures, and Clinical Outcomes

Baseline data from each modality of interest, i.e., PET, EEG, fMRI, MRI, cognitive measures, peripheral molecular markers, and clinical/demographic patient profiles, will be available for evaluating associations with the clinical outcomes for the entire group (n = 100 included). Changes from baseline data will be available for the subgroup (around n = 40 invited), who will be re-examined with brain scans and EEG for evaluating an association between changed measures and clinical outcomes. Similarly, cognitive follow-up data will be collected for all patients after 12 weeks of treatment. Primary analyses will test mean differences in baseline measures of the biomarkers from each modality between healthy controls and patients as well as response groups (remitter vs. non-responder at week 8, i.e., primary clinical outcome) using multiple linear regressions. This analysis focuses on the two extreme outcome groups. Secondary analyses will test the association between baseline measures of the biomarkers from each modality and antidepressant treatment response on a continuous scale, i.e., relative change in HAMD_6_, using linear multiple regression. This analysis incorporates the full spectrum of clinical outcomes. Similar analyses will be performed to study the association between the change from baseline measures of the biomarkers and the clinical outcomes. Regression models will be adjusted for age and sex, as well as modality-specific relevant covariates. For instance, 5-HTTLPR status is predictive of 5-HT_4_R binding (70) and will be adjusted for in the analyses concerning 5-HT_4_R. When relevant, interactions will be evaluated, e.g., we will test if the association between the clinical outcome and 5-HT_4_R is moderated by inflammatory status. Diagnostic regression tools will be used to assess model’s assumptions (e.g., linearity of the effects, normality assumption for residuals). When violated, corrective procedures will be used (e.g., splines and bootstrap resampling) (113). As appropriate, adjustments for multiple comparisons will be performed within each modality. In the analysis of the PET data, we will instead use a Latent Variable Model relating the 5-HT_4_R binding in several brain regions (neocortex, caudate nucleus, putamen and hippocampus) to treatment outcome *via* a latent variable (114). This allows us to assess the association between 5-HT_4_R binding and clinical outcome with a single test. Patients are considered with un-verified compliance if they have taken less than 2/3 of their antidepressant medicine, missed their week 8 visit, or have undetectable serum drug levels at week 8 (i.e., <10 nM for escitalopram and <15 nM for duloxetine). Patients with un-verified compliance will not be included in primary longitudinal analyses of treatment response. Missing data will therefore be handled using complete case analysis which in regression models is valid when the probability of dropping out of the study is, conditional on the covariates, independent of the outcome. If any participants were to be excluded during the study because of their clinical outcome, a sensitivity analysis will be performed.

#### Evaluation of the Predictive Value of the Biomarkers Within Modality

Logistic regression models for the primary clinical outcome will be used to obtain the probability of each patient to be a remitter (vs. a non-responder) based on its clinical data and the value of a modality-specific biomarker. Given a threshold (e.g., 0.5), patients with an estimated probability greater than the threshold will be predicted to be remitters, otherwise to be non-responders. The receiver operating characteristic (ROC) curve will be used to assess the compromise between sensitivity and specificity of this classification across thresholds. Since a 33% remission rate is expected in treatment regimen comparable to ours (5), we will focus on the ROC curve with high-specificity. The AUC (area under the curve) of the relevant part of the ROC curve will be reported as a summary of the predictive performance of each biomarker. The classification performance (accuracy, positive predictive value, negative predictive value) at the threshold optimizing the sum specificity and specificity will also be reported. To limit optimistic biases, these measures will be estimated using five-fold cross-validation (115). A permutation procedure will be used to obtain the null distribution of the predictive performance, against which the observed performance will be compared. Additional classification schemes may be considered (e.g., responder status as defined by ≥50% reduction in HAMD_6_ at week 8), with appropriate adjustment for inflated type-I error, to facilitate comparison of the current data with other relevant clinical trials. The predictor performance will be evaluated in a modality specific fashion and at a next stage, combined predictors will be evaluated.

#### Predictive Value of the Biomarkers Across Modalities

Two strategies will be considered to optimize prediction of treatment response using biomarkers measured at baseline across modalities. In the first strategy, we will combine the specific biomarker-candidates across all modalities (as predefined in **Appendix 1**), which will generate around 50 candidate biomarkers. A dimension reduction step will be used to define a small number of predictors (roughly 5–10) that will be used in a logistic regression model. The second strategy will use an algorithm to (i) identify, in a data-driven way, biomarkers with a predictive value among all the existing biomarkers (roughly 5,000–10,000) and (ii) predict treatment response based on the identified subset of informative biomarkers. We will investigate the use of machine learning methods (e.g., random forest, neural networks) as well as ensemble methods [e.g., Super Learner (116)]. The assessment of the predictive performance of these strategies will be carried out as described in the previous section.

### Ethics Approval and Consent to Participate

The study protocol complies with the Declaration of Helsinki II and data collected during the trial will be monitored throughout the study period (for every 10^th^ patient included) by an independent Good Clinical Practice unit in the capital region of Denmark (www.gcp-enhed.dk/en). The study has been approved by the Committees on Health Research Ethics in the Capital Region of Denmark (reference number: H-15017713), the Danish Data Protection Agency (04711/RH-2016-163), and Danish Medicines Agency (protocol number: NeuroPharm-NP1, EudraCT-number 2016-001626-34). All potential participants will receive oral and written information about the study by the enrolling clinician, and all enrolled participants will provide written informed consent prior to inclusion. Adverse events have been scheduled to be reported annually to the Danish Medicines Agency. The study was registered at clinicaltrials.gov prior to initiation (NCT02869035), date: 08.16.2016.

### Availability of Data and Materials

Data management and monitoring during the study agrees to the rules on protection of personal data. To protect confidentiality, paper-based material (e.g., cognitive test results) will be stored in a secured archive, while electronical data files that are identifiable will be stored in password secured files behind firewall in accordance to regulations. To promote data quality, the primary outcome measurement (HAMD_17/6_ scores) will be obtained during interviews on paper, manually transferred into the local database through LimeSurvey and cross-checked twice before used in analyses. Biological material will be coded with a unique identification-number and access to de-identification keys is restricted to authorized personnel only and stored in a temporary biobank located in secured areas in the laboratory facility. The biomaterial will later be analyzed in batches to reduce noise, and potential extra material after the end of the clinical trial will be transferred to the CIMBI biobank (71). All biological material will ultimately be anonymized after 15 years after the end of trial.

### Progress to Date

The study opened for inclusion of patients in August 2016. To date, the remaining biological data including genetic status of healthy controls are planned to be collected. Obtained biological material is currently being analyzed and processing of imaging data is on-going. Results from the trial are planned to be communicated to the participants and public through publication in international medical journals.

## Discussion

The main purpose of the present study is to identify individual or combined predictors (biomarkers) of standard pharmacological antidepressant treatment outcome in MDD, by using multiple modalities such as brain imaging (PET, fMRI), EEG, cognitive tools, and clinical and molecular markers. Special emphasis in the study design has been given to evaluate the biomarker 5-HT_4_R PET as a promising clinically relevant tool since the 5HT_4_R availability is of interest in the pathophysiology and as a therapeutic target in MDD, and also as an index of serotonin tonus. The aim of this trial is not to investigate the specific treatment efficacy but to investigate biomarkers for response to standard treatment in a naturalistic setting, e.g., similar to the STAR*D trial (117). The study includes multiple cross-sectional and longitudinal measures in a large number of patients and controls, which offers a unique opportunity to (a) uncover potential biomarkers or clusters of biomarkers of treatment prediction, (b) apply the findings for stratification of MDD, (c) advance the understanding of pathophysiological underpinnings of MDD, (d) map neurobiological signatures of antidepressant treatment response, and lastly (e) to ideally pave a way for a precision medicine approach for optimized treatment of MDD.

## Ethics Statement

The studies involving human participants were reviewed and approved by the Committees on Health Research Ethics in the Capital Region of Denmark (reference number: H-15017713). The patients/participants provided their written informed consent to participate in this study.

## Author Contributions

GK, MJ, and VF conceived the concept of the study, with help and support from AJ and KK-F. KK-F, C-TI, VD, DS, PF, MG, HP, AG, and BO have supervised study design and contributed to drafting of the manuscript. All authors contributed to the article and approved the submitted version.

## Funding

Economic support for the study was granted from the Innovation Fund Denmark (GrantID: 5189-00087A), Research Fund of the Mental Health Services - Capital Region of Denmark, Savværksejer Jeppe Juhl og hustru Ovita Juhls Mindelegat, Augustinus Foundation (GrantID: 16-0058), Research Council of Rigshospitalet the independent research fund Denmark (GrantID: DFF-6120-00038), and the Lundbeck Foundation. H. Lundbeck A/S had no influence on study design and will not be involved in data processing or in publishing the results of the trial.

## Conflict of Interest

C-TI was partly employed by company H. Lundbeck A/S. VF and GK have served as consultants for SAGE therapeutics, GK has served as a speaker in a Janssen sponsored symposium. MJ and VF has given talks sponsored by Lundbeck Pharma and MJ for Boehringer Ingelheim.

The remaining authors declare that the research was conducted in the absence of any commercial or financial relationships that could be construed as a potential conflict of interest.

## Abbreviations

MDD, Major Depressive Disorder; 5-HT: 5-hydroxytryptamine (Serotonin); SSRI, Selective serotonin reuptake inhibitor; SERT, Serotonin transporter; PET, Positron emission tomography; fMRI, Functional magnetic resonance imaging; EEG, Electroencephalogram; 5-HT_4_R, Serotonin 4 receptor; rs-fMRI, Resting state functional magnetic resonance imaging; ERP, Event-related potential; LDAEP, Loudness dependence of auditory evoked potentials; CAR, Cortisol awakening response; HPA-axis, Hypothalamic-pituitary-adrenal axis; HAMD_17_, 17-item Hamilton Depression Rating Scale; HAMD_6_, 6-item Hamilton Depression Rating Scale.

## References

[1] Global Burden of Disease Study 2013 Collaborators Global, regional, and national incidence, prevalence, and years lived with disability for 301 acute and chronic diseases and injuries in 188 countries, 1990-2013: a systematic analysis for the Global Burden of Disease Study 2013. Lancet (London, England) 386(9995):743–800. 10.1016/S0140-6736(15)60692-4 PMC4561509 26063472

[2] Ferrari AJ Charlson FJ Norman RE Patten SB Freedman G Murray C Burden of Depressive Disorders by Country, Sex, Age, and Year: Findings from the Global Burden of Disease Study 2010. PloS Med (2013) 10(11):e1001547. 10.1371/journal.pmed.10015473 24223526 PMC3818162

[3] Benmansour S Owens WA Cecchi M Morilak DA Frazer A Serotonin Clearance In Vivo Is Altered to a Greater Extent by Antidepressant-Induced Downregulation of the Serotonin Transporter than by Acute Blockade of this Transporter. J Neurosci (2002) 22(15):6766–72. 10.1523/jneurosci.22-15-06766.20024 PMC6758131 12151556

[4] Smith DF Jakobsen S Molecular neurobiology of depression: PET findings on the elusive correlation with symptom severity. Front Psychiatry (2013) 4:8. 10.3389/fpsyt.2013.00008 PMC3586775 23459670

[5] Rush AJ Trivedi MH Wisniewski SR Nierenberg AA Stewart JW Warden D Acute and longer-term outcomes in depressed outpatients requiring one or several treatment steps: A STAR*D report. Am J Psychiatry (2006) 163(11):1905–17. 10.1176/ajp.2006.163.11.19056 17074942

[6] Nakajima S Uchida H Suzuki T Watanabe K Hirano J Yagihashi T Is switching antidepressants following early nonresponse more beneficial in acute-phase treatment of depression?: A randomized open-label trial. Prog Neuropsychopharmacol Biol Psychiatry (2011) 35(8):1983–9. 10.1016/j.pnpbp.2011.08.008 21889560

[7] Hasler G Pathophysiology of depression: Do we have any solid evidence of interest to clinicians? World Psychiatry (2010) 9(3):155–61. 10.1002/j.2051-5545.2010.tb00298.x PMC2950973 20975857

[8] Hellwig S Domschke K Update on PET imaging biomarkers in the diagnosis of neuropsychiatric disorders. Curr Opin Neurol (2019) 32(4):539–47. 10.1097/wco.0000000000000705 31083013

[9] Strawbridge R Young AH Cleare AJ Biomarkers for depression: Recent insights, current challenges and future prospects. Neuropsychiatr Dis Treat (2017) 13:1245–62. 10.2147/NDT.S114542 PMC5436791 28546750

[10] Williams LM Rush AJ Koslow SH Wisniewski SR Cooper NJ Nemeroff C International Study to Predict Optimized Treatment for Depression (iSPOT-D), a randomized clinical trial: Rationale and protocol. Trials (2011) 12:4. 10.1186/1745-6215-12-4 21208417 PMC3036635

[11] Trivedi MH McGrath PJ Fava M Parsey RV Kurian BT Phillips ML Establishing moderators and biosignatures of antidepressant response in clinical care (EMBARC): Rationale and design. J Psychiatr Res (2016) 78:11–23. 10.1016/j.jpsychires.2016.03.001 27038550 PMC6100771

[12] Lam RW Milev R Rotzinger S Andreazza AC Blier P Brenner C Discovering biomarkers for antidepressant response: Protocol from the Canadian biomarker integration network in depression (CAN-BIND) and clinical characteristics of the first patient cohort. BMC Psychiatry (2016) 16:105. 10.1186/s12888-016-0785-x 27084692 PMC4833905

[13] Gong B Naveed S Hafeez DM Afzal KI Majeed S Abele J Neuroimaging in Psychiatric Disorders: A Bibliometric Analysis of the 100 Most Highly Cited Articles. J Neuroimaging (2019) 29(1):14–33. 10.1111/jon.12570 30311320

[14] Rebholz H Friedman E Castello J Alterations of Expression of the Serotonin 5-HT4 Receptor in Brain Disorders. Int J Mol Sci (2018) 19(11): 3581. 10.3390/ijms19113581 PMC6274737 30428567

[15] Lucas G Rymar VV Du J Mnie-Filali O Bisgaard C Manta S Serotonin(4) (5-HT(4)) receptor agonists are putative antidepressants with a rapid onset of action. Neuron (2007) 55(5):712–25. 10.1016/j.neuron.2007.07.041 17785179

[16] Licht CL Marcussen AB Wegener G Overstreet DH Aznar S Knudsen GM The brain 5-HT4 receptor binding is down-regulated in the Flinders Sensitive Line depression model and in response to paroxetine administration. J Neurochem (2009) 109(5):1363–74. 10.1111/j.1471-4159.2009.06050.x 19476548

[17] Vidal R Valdizán EM Mostany R Pazos A Castro E Long-term treatment with fluoxetine induces desensitization of 5-HT 4 receptor-dependent signalling and functionality in rat brain. J Neurochem (2009) 110(3):1120–7. 10.1111/j.1471-4159.2009.06210.x 19522734

[18] Haahr ME Fisher PM Jensen CG Frokjaer VG Mc Mahon B Madsen K Central 5-HT4 receptor binding as biomarker of serotonergic tonus in humans: A [11C]SB207145 PET study. Mol Psychiatry (2014) 19(4):427–32. 10.1038/mp.2013.147 24189342

[19] Stuhrmann A Suslow T Dannlowski U Facial emotion processing in major depression: A systematic review of neuroimaging findings. Biol Mood Anxiety Disord (2011) 1(1):10. 10.1186/2045-5380-1-10 22738433 PMC3384264

[20] Williams LM Korgaonkar MS Song YC Paton R Eagles S Goldstein-Piekarski A Amygdala Reactivity to Emotional Faces in the Prediction of General and Medication-Specific Responses to Antidepressant Treatment in the Randomized iSPOT-D Trial. Neuropsychopharmacology (2015) 40(10):2398–408. 10.1038/npp.2015.89 PMC4538354 25824424

[21] Goldstein-Piekarski AN Korgaonkar MS Green E Suppes T Schatzberg AF Hastie T Human amygdala engagement moderated by early life stress exposure is a biobehavioral target for predicting recovery on antidepressants. Proc Natl Acad Sci (2016) 113(42):11955–60. 10.1073/pnas.1606671113 PMC5081583 27791054

[22] Fisher PMD Haahr ME Jensen CG Frokjaer VG Siebner HR Knudsen GM Fluctuations in [11C]SB207145 PET Binding Associated with Change in Threat-Related Amygdala Reactivity in Humans. Neuropsychopharmacology (2015) 40(6):1510–8. 10.1038/npp.2014.339 PMC4397409 25560201

[23] Fisher PM Hariri AR Linking variability in brain chemistry and circuit function through multimodal human neuroimaging. Genes Brain Behav (2012) 11(6):633–42. 10.1111/j.1601-183X.2012.00786.x 22443230

[24] Raichle ME The Brain’s Default Mode Network. Annu Rev Neurosci (2015) 38:433–47. 10.1146/annurev-neuro-071013-014030 25938726

[25] Kaiser RH Andrews-Hanna JR Wager TD Pizzagalli DA Large-scale network dysfunction in major depressive disorder: A meta-analysis of resting-state functional connectivity. JAMA Psychiatry (2015). 72(6):603–11. 10.1001/jamapsychiatry.2015.0071 PMC4456260 25785575

[26] Drysdale AT Grosenick L Downar J Dunlop K Mansouri F Meng Y Resting-state connectivity biomarkers define neurophysiological subtypes of depression. Nat Med (2017) 23(1):28–38. 10.1038/nm.4246 27918562 PMC5624035

[27] Dinga R Schmaal L Penninx BWJH van Tol MJ Veltman DJ van Velzen L Evaluating the evidence for biotypes of depression: Methodological replication and extension of. NeuroImage Clin (2019) 22:101796 10.1016/j.nicl.2019.101796 30935858 PMC6543446

[28] Patel MJ Khalaf A Aizenstein HJ Studying depression using imaging and machine learning methods. NeuroImage Clin (2016) 10:115–23. 10.1016/j.nicl.2015.11.003 PMC4683422 26759786

[29] Olbrich S Arns M EEG biomarkers in major depressive disorder: Discriminative power and prediction of treatment response. Int Rev Psychiatry (2013) 25(5):604–18. 10.3109/09540261.2013.816269 24151805

[30] Arns M Bruder G Hegerl U Spooner C Palmer DM Etkin A EEG alpha asymmetry as a gender-specific predictor of outcome to acute treatment with different antidepressant medications in the randomized iSPOT-D study. Clin Neurophysiol (2016) 127(1):509–19. 10.1016/j.clinph.2015.05.032 26189209

[31] Pizzagalli DA Frontocingulate dysfunction in depression: Toward biomarkers of treatment response. Neuropsychopharmacology (2011) 36(1):183–206. 10.1038/npp.2010.166 20861828 PMC3036952

[32] Pizzagalli DA Webb CA Dillon DG Pretreatment rostral anterior cingulate cortex theta activity in relation to symptom improvement in depression: A randomized clinical trial. JAMA Psychiatry (2018) 75(6):547–54. 10.1001/jamapsychiatry.2018.0252 PMC6083825 29641834

[33] Jaworska N Protzner A Electrocortical features of depression and their clinical utility in assessing antidepressant treatment outcome. Can J Psychiatry (2013) 58(9):509–14. 10.1177/070674371305800905 24099498

[34] Mulert C Juckel G Brunnmeier M Karch S Leicht G Mergl R Prediction of treatment response in major depression: Integration of concepts. J Affect Disord (2007) 98(3):215–25. 10.1016/j.jad.2006.07.021 16996140

[35] Juckel G Pogarell O Augustin H Mulert C Müller-Siecheneder F Frodl T Differential prediction of first clinical response to serotonergic and noradrenergic antidepressants using the loudness dependence of auditory evoked potentials in patients with major depressive disorder. J Clin Psychiatry (2007) 68(8):1206–12. 10.4088/JCP.v68n0806 17854244

[36] Rock PL Roiser JP Riedel WJ Blackwell AD Cognitive impairment in depression: A systematic review and meta-analysis. Psychol Med (2014) 44(10):2029–40. 10.1017/S0033291713002535 24168753

[37] Weightman MJ Knight MJ Baune BT A systematic review of the impact of social cognitive deficits on psychosocial functioning in major depressive disorder and opportunities for therapeutic intervention. Psychiatry Res (2019) 274:195–212. 10.1016/j.psychres.2019.02.035 30807971

[38] Roiser JP Elliott R Sahakian BJ Cognitive mechanisms of treatment in depression. Neuropsychopharmacology (2012) 37(1):117–36. 10.1038/npp.2011.183 PMC3238070 21976044

[39] Kingslake J Dias R Dawson GR Simon J Goodwin GM Harmer CJ The effects of using the PReDicT Test to guide the antidepressant treatment of depressed patients: Study protocol for a randomised controlled trial. Trials (2017) 18(1):558. 10.1186/s13063-017-2247-2 29169399 PMC5701462

[40] Zuckerman H Pan Z Park C Brietzke E Musial N Shariq AS Recognition and Treatment of Cognitive Dysfunction in Major Depressive Disorder. Front Psychiatry (2018) 9:655. 10.3389/fpsyt.2018.00655 30564155 PMC6288549

[41] Haahr ME Fisher P Holst K Madsen K Jensen CG Marner L The 5-HT4 receptor levels in hippocampus correlates inversely with memory test performance in humans. Hum Brain Mapp (2013) 34(11):3066–74. 10.1002/hbm.22123 PMC6869972 22736538

[42] Stenbæk DS Fisher PM Ozenne B Andersen E Hjordt LV McMahon B Brain serotonin 4 receptor binding is inversely associated with verbal memory recall. Brain Behav (2017) 7(4):e00674. 10.1002/brb3.674 28413715 PMC5390847

[43] Dantzer R O’Connor JC Lawson MA Kelley KW Inflammation-associated depression: From serotonin to kynurenine. Psychoneuroendocrinology (2011) 36(3):426–36. 10.1016/j.psyneuen.2010.09.012 PMC3053088 21041030

[44] Lanquillon S Krieg JC Bening-Abu-Shach U Vedder H Cytokine production and treatment response in major depressive disorder. Neuropsychopharmacology (2000) 22(4):370–9. 10.1016/S0893-133X(99)00134-7 10700656

[45] Liu JJ Bin WY Strawbridge R Bao Y Chang S Shi L Peripheral cytokine levels and response to antidepressant treatment in depression: a systematic review and meta-analysis. Mol Psychiatry (2019) 25(2):339–50. 10.1038/s41380-019-0474-5 31427752

[46] Richards EM Zanotti-Fregonara P Fujita M Newman L Farmer C Ballard ED PET radioligand binding to translocator protein (TSPO) is increased in unmedicated depressed subjects. EJNMMI Res (2018) 8(1):57. 10.1186/s13550-018-0401-9 29971587 PMC6029989

[47] Zhu CB Blakely RD Hewlett WA The proinflammatory cytokines interleukin-1beta and tumor necrosis factor-alpha activate serotonin transporters. Neuropsychopharmacology (2006) 31(10):2121–31. 10.1038/sj.npp.1301029 16452991

[48] Allison DJ Ditor DS The common inflammatory etiology of depression and cognitive impairment: A therapeutic target. J Neuroinflamm (2014) 11:151 10.1186/s12974-014-0151-1 PMC4156619 25178630

[49] Khan A Faucett J Morrison S Brown WA Comparative mortality risk in adult patients with schizophrenia, depression, bipolar disorder, anxiety disorders, and attention-deficit/ hyperactivity disorder participating in psychopharmacology clinical trials. JAMA Psychiatry (2013) 70(10):1091–9. 10.1001/jamapsychiatry.2013.149 23986353

[50] Laursen TM Musliner KL Benros ME Vestergaard M Munk-Olsen T Mortality and life expectancy in persons with severe unipolar depression. J Affect Disord (2016) 193:203–7. 10.1016/j.jad.2015.12.067 26773921

[51] Wolkowitz OW Epel ES Reus VI Mellon SH Depression gets old fast: Do stress and depression accelerate cell aging? Depress Anxiety (2010) 27(4):327–38. 10.1002/da.20686 20376837

[52] Jorgensen A Maigaard K Wörtwein G Hageman I Henriksen T Weimann A Chronic restraint stress in rats causes sustained increase in urinary corticosterone excretion without affecting cerebral or systemic oxidatively generated DNA/RNA damage. Prog Neuropsychopharmacol Biol Psychiatry (2013) 40:30–7. 10.1016/j.pnpbp.2012.08.016 22960608

[53] Jørgensen A Brødbaek K Weimann A Fink-Jensen A Knorr U Greisen Soendergaard M Henriksen T Jørgensen: Increased Systemic Oxidatively Generated DNA and RNA Damage in Schizophrenia. Psychiatry Research 2013 (Epub ahead of print). Dan Med J (2013) 209(3):417–23. 10.1016/j.psychres.2013.01.033 23465294

[54] Joergensen A Broedbaek K Weimann A Semba RD Ferrucci L Joergensen MB Association between urinary excretion of cortisol and markers of oxidatively damaged DNA and RNA in humans. PloS One (2011) 6(6):e20795. 10.1371/journal.pone.0020795 21687734 PMC3110199

[55] Jorgensen A Krogh J Miskowiak K Bolwig TG Kessing LV Fink-Jensen A Systemic oxidatively generated DNA/RNA damage in clinical depression: Associations to symptom severity and response to electroconvulsive therapy. J Affect Disord (2013) 149(1-3):355–62. 10.1016/j.jad.2013.02.011 23497793

[56] Walton NM Shin R Tajinda K Heusner CL Kogan JH Miyake S Adult neurogenesis transiently generates oxidative stress. PloS One (2012) 7(4):e35264. 10.1371/journal.pone.0035264 22558133 PMC3340368

[57] Chung CP Schmidt D Stein CM Morrow JD Salomon RM Increased oxidative stress in patients with depression and its relationship to treatment. Psychiatry Res (2012) 206(2-3):213–6. 10.1016/j.psychres.2012.10.018 PMC3615036 23245537

[58] Jørgensen A Oxidatively generated DNA/RNA damage in psychological stress states. Dan Med J (2013) 60(7):1–14. 23809980

[59] Da Cunha-Bang S Hjordt LV Dam VH Stenbæk DS Sestoft D Knudsen GM Anterior cingulate serotonin 1B receptor binding is positively associated with inhibitory control and amygdala reactivity to aversive faces. Eur Neuropsychopharmacol (2016) 92:199–204. 10.1016/s0924-977x(16)31246-9

[60] Fava M Rush AJ Alpert JE Balasubramani GK Wisniewski SR Carmin CN Difference in treatment outcome in outpatients with anxious versus nonanxious depression: A STAR*D report. Am J Psychiatry (2008) 165(3):342–51. 10.1176/appi.ajp.2007.06111868 18172020

[61] Montejo AL Montejo L Baldwin DS The impact of severe mental disorders and psychotropic medications on sexual health and its implications for clinical management. World Psychiatry (2018) 17(1):3–11. 10.1002/wps.20509 29352532 PMC5775119

[62] Williams VSL Edin HM Hogue SL Fehnel SE Baldwin DS Prevalence and impact of antidepressant-associated sexual dysfunction in three European countries: Replication in a cross-sectional patient survey. J Psychopharmacol (2010) 24(4):489–96. 10.1177/0269881109102779 19329551

[63] Pfaus JG Pathways of sexual desire. J Sex Med (2009) 6(6):1506–33. 10.1111/j.1743-6109.2009.01309.x 19453889

[64] Jakobsen GR Fisher PM Dyssegaard A McMahon B Holst KK Lehel S Brain serotonin 4 receptor binding is associated with the cortisol awakening response. Psychoneuroendocrinology (2016) 67:124–32. 10.1016/j.psyneuen.2016.01.032 26894483

[65] Frokjaer VG Erritzoe D Holst KK Jensen PS Rasmussen PM Fisher PM Prefrontal serotonin transporter availability is positively associated with the cortisol awakening response. Eur Neuropsychopharmacol (2013) 23(4):285–94. 10.1016/j.euroneuro.2012.05.013 22732516

[66] Vrshek-Schallhorn S Doane LD Mineka S Zinbarg RE Craske MG Adam EK The cortisol awakening response predicts major depression: Predictive stability over a 4-year follow-up and effect of depression history. Psychol Med (2013) 43(3):483–93. 10.1017/S0033291712001213 PMC3500423 22652338

[67] Booij SH Bouma EMC De Jonge P Ormel J Oldehinkel AJ Chronicity of depressive problems and the cortisol response to psychosocial stress in adolescents: The TRAILS study. Psychoneuroendocrinology (2013) 38(5):659–66. 10.1016/j.psyneuen.2012.08.004 22963816

[68] Ruhé HG Khoenkhoen SJ Ottenhof KW Koeter MW Mocking RJT Schene AH Longitudinal effects of the SSRI paroxetine on salivary cortisol in Major Depressive Disorder. Psychoneuroendocrinology (2015) 52:261–71. 10.1016/j.psyneuen.2014.10.024 25544738

[69] Timmerby N Andersen JH Søndergaard S Østergaard SD Bech P Bech P. A Systematic Review of the Clinimetric Properties of the 6-Item Version of the Hamilton Depression Rating Scale (HAM-D6). Psychother Psychosom (2017) 86(3):141–9. 10.1159/000457131 28490031

[70] Fisher PM Holst KK Mc Mahon B Haahr ME Madsen K Gillings N 5-HTTLPR status predictive of neocortical 5-HT 4 binding assessed with [11C]SB207145 PET in humans. Neuroimage (2012) 62(1):130–6. 10.1016/j.neuroimage.2012.05.013 22584237

[71] Knudsen GM Jensen PS Erritzoe D Baaré W Ettrup A Fisher PM The Center for Integrated Molecular Brain Imaging (Cimbi) database. Neuroimage (2016) 124(Pt B):1213–9. 10.1016/j.neuroimage.2015.04.025 25891375

[72] Sheehan DV Lecrubier Y Sheehan KH Amorim P Janavs J Weiller E The Mini-International Neuropsychiatric Interview (M.I.N.I.): The development and validation of a structured diagnostic psychiatric interview for DSM-IV and ICD-10. In: J Clin Psychiatry (1998) 50(Suppl 20):22–57. 10.1016/S0924-9338(99)80239-9 9881538

[73] Olsen LR Mortensen EL Bech P Prevalence of major depression and stress indicators in the Danish general population. Acta Psychiatr Scand (2004) 109(2):96–103. 10.1046/j.0001-690X.2003.00231.x 14725589

[74] Culpepper L Escitalopram: A New SSRI for the Treatment of Depression in Primary Care. Prim Care Companion J Clin Psychiatry (2002) 4(6):209–14. 10.4088/PCC.v04n0601 PMC315490 15014711

[75] Bushnell GA Stürmer T Gaynes BN Pate V Miller M Simultaneous antidepressant and benzodiazepine new use and subsequent long-term benzodiazepine use in adults with depression, United States, 2001-2014. JAMA Psychiatry (2017) 74(7):747–55. 10.1001/jamapsychiatry.2017.1273 PMC5710248 28593281

[76] Marner L Gillings N Madsen K Erritzoe D Baaré WF Svarer C Brain imaging of serotonin 4 receptors in humans with [11C]SB207145-PET. Neuroimage (2010) 50(3):855–61. 10.1016/j.neuroimage.2010.01.054 20096787

[77] Bymaster FP Dreshfield-Ahmad LJ Threlkeld PG Shaw JL Thompson L Nelson DL Comparative affinity of duloxetine and venlafaxine for serotonin and norepinephrine transporters in vitro and in vivo, human serotonin receptor subtypes, and other neuronal receptors. Neuropsychopharmacology (2001) 25(6):871–80. 10.1016/S0893-133X(01)00298-6 11750180

[78] Lingjærde O Ahlfors UG Bech P Dencker SJ Elgen K The UKU side effect rating scale: A new comprehensive rating scale for psychotropic drugs and a cross-sectional study of side effects in neuroleptic-treated patients. Acta Psychiatr Scand (1987) 334:1–100. 10.1111/j.1600-0447.1987.tb10566.x 2887090

[79] Marner L Gillings N Comley RA Baaré WF Rabiner EA Wilson AA Kinetic Modeling of 11C-SB207145 Binding to 5-HT4 Receptors in the Human Brain In Vivo. J Nucl Med (2009) 50(6):900–8. 10.2967/jnumed.108.058552 19470850

[80] Sureau FC Reader AJ Comtat C Leroy C Ribeiro MJ Buvat I Impact of Image-Space Resolution Modeling for Studies with the High-Resolution Research Tomograph. J Nucl Med (2008) 49(6):1000–8. 10.2967/jnumed.107.045351 18511844

[81] Keller SH Svarer C Sibomana M Attenuation correction for the HRRT PET-scanner using transmission scatter correction and total variation regularization. IEEE Trans Med Imaging (2013) 32(9):1611–21. 10.1109/TMI.2013.2261313 23661313

[82] Woods RP Cherry SR Mazziotta JC Rapid automated algorithm for aligning and reslicing pet images. J Comput Assist Tomogr (1992) 16(4):620–33. 10.1097/00004728-199207000-00024 1629424

[83] Svarer C Madsen K Hasselbalch SG Pinborg LH Haugbøl S Frøkjaer VG MR-based automatic delineation of volumes of interest in human brain PET images using probability maps. Neuroimage (2005) 24(4):969–79. 10.1016/j.neuroimage.2004.10.017 15670674

[84] Madsen K Marner L Haahr M Gillings N Knudsen GM Mass dose effects and in vivo affinity in brain PET receptor studies - a study of cerebral 5-HT 4 receptor binding with [11C]SB207145. Nucl Med Biol (2011) 38(8):1085–91. 10.1016/j.nucmedbio.2011.04.006 21831646

[85] da Cunha-Bang S Fisher PM Hjordt LV Perfalk E Persson Skibsted A Bock C Violent offenders respond to provocations with high amygdala and striatal reactivity. Soc Cognit Affect Neurosci (2017) 12(5):802–10. 10.1093/scan/nsx006 PMC5460055 28338916

[86] Nikolova YS Iruku SP Lin C-W Conley E D Puralewski R French B FRAS1-related extracellular matrix 3 (FREM3) single-nucleotide polymorphism effects on gene expression, amygdala reactivity and perceptual processing speed: An accelerated aging pathway of depression risk. Front Psychol (2015) 6:1377. 10.3389/fpsyg.2015.01377 26441752 PMC4584966

[87] Forbes EE Hariri AR Martin SL Silk JS Moyles DL Fisher PM Altered striatal activation predicting real-world positive affect in adolescent major depressive disorder. Am J Psychiatry (2009) 166(1):64–73. 10.1176/appi.ajp.2008.07081336 19047324 PMC2701209

[88] Nikolova YS Ferrell RE Manuck SB Hariri AR Multilocus genetic profile for dopamine signaling predicts ventral striatum reactivity. Neuropsychopharmacology (2011) 36(9):1940–7. 10.1038/npp.2011.82 PMC3154113 21593733

[89] Bland AR Roiser JP Mehta MA Schei T Boland H Campbell-Meiklejohn DK EMOTICOM: A Neuropsychological Test Battery to Evaluate Emotion, Motivation, Impulsivity, and Social Cognition. Front Behav Neurosci (2016) 10:25. 10.3389/fnbeh.2016.00025 26941628 PMC4764711

[90] Jensen CG Hjordt LV Stenbæk DS Andersen E Back SK Lansner J Development and psychometric validation of the verbal affective memory test. Memory (2016) 24(9):1208–23. 10.1080/09658211.2015.1087573 26401886

[91] Costa P McCrae R (2008). The revised NEO personality inventory (NEO-PI-R). The SAGE Handbook of Personality Theory and Assessment. 2:179–98. 10.4135/9781849200479.n9

[92] Sanders B Becker-Lausen E The measurement of psychological maltreatment: Early data on the child abuse and trauma scale. Child Abus Negl (1995) 19(3):315–23. 10.1016/S0145-2134(94)00131-6 9278731

[93] Harrison EL Baune BT Modulation of early stress-induced neurobiological changes: A review of behavioural and pharmacological interventions in animal models. Transl Psychiatry (2014) 4(5):e390. 10.1038/tp.2014.31 24825729 PMC4035722

[94] Oldfield RC The assessment and analysis of handedness: The Edinburgh inventory. Neuropsychologia (1971) 9(1):97–113. 10.1016/0028-3932(71)90067-4 5146491

[95] Parker G Tupling H Brown LB. A Parental Bonding Instrument. Br J Med Psychol (1979) 52:1–10. 10.1111/j.2044-8341.1979.tb02487.x

[96] McNair DM Lorr M Droppleman LF Revised manual for the Profile of Mood States. (San Diego, CA: Educational and Industrial Testing Services) (1992).

[97] Beck AT Steer RA Brown GK Manual for the Beck depression inventory-II. San Antonio TX Psychol Corp (1996) 10.1037/t00742-000

[98] Forsell Y The Major Depression Inventory versus schedules for clinical assessment in neuropsychiatry in a population sample. Soc Psychiatry Psychiatr Epidemiol (2005) 40(3):209–13. 10.1007/s00127-005-0876-3 15742226

[99] Cohen S Kamarck T Mermelstein R A global measure of perceived stress. J Health Soc Behav (1983) 24(4):385–96. 10.2307/2136404 6668417

[100] Cohen S Williamson G Perceived stress in a probability sample of the United States. Soc Psychol Heal (1988) 31–67. 10.1111/j.1559-1816.1983.tb02325.x

[101] Snaith RP Hamilton M Morley S Humayan A Hargreaves D Trigwell P A scale for the assessment of hedonic tone. The Snaith-Hamilton Pleasure Scale. Br J Psychiatry (1995) 167(1):99–103. 10.1192/bjp.167.1.99 7551619

[102] Treynor W Gonzalez R Nolen-Hoeksema S Rumination reconsidered: A psychometric analysis. Cognit Ther Res (2003) 27:247–59. 10.1023/A:1023910315561

[103] Clayton AH McGarvey EL Clavet GJ The changes in sexual functioning questionnaire (CSFQ): Development, reliability, and validity. Psychopharmacol Bull (1997) 33(4):731–45. 9493486

[104] Eplov L Giraldi A Davidsen M Garde K Kamper-Jørgensen F Sexual desire in a nationally representative danish population. J Sex Med (2007) 4(1):47–56. 10.1111/j.1743-6109.2006.00396.x 17233775

[105] Bech P Rating scales in depression: Limitations and pitfalls. Dialogues Clin Neurosci (2006) 8(2):207–15. 10.31887/DCNS.2006.8.2/pbech PMC3181766 16889106

[106] Dahl J Ormstad H Aass HCD Malt UF Bendz LT Sandvik L The plasma levels of various cytokines are increased during ongoing depression and are reduced to normal levels after recovery. Psychoneuroendocrinology (2014) 45:77–86. 10.1016/j.psyneuen.2014.03.019 24845179

[107] Dowlati Y Herrmann N Swardfager W Liu H Sham L Reim EK A Meta-Analysis of Cytokines in Major Depression. Biol Psychiatry (2010) 67(5):446–57. 10.1016/j.biopsych.2009.09.033 20015486

[108] Howren MB Lamkin DM Suls J Associations of depression with c-reactive protein, IL-1, and IL-6: A meta-analysis. Psychosom Med (2009). 71(2):171–86. 10.1097/PSY.0b013e3181907c1b 19188531

[109] Liu Y Ho RCM Mak A Interleukin (IL)-6, tumour necrosis factor alpha (TNF-α) and soluble interleukin-2 receptors (sIL-2R) are elevated in patients with major depressive disorder: A meta-analysis and meta-regression. J Affect Disord (2012). 139(3):230–9. 10.1016/j.jad.2011.08.003 21872339

[110] Tammiste A Jiang T Fischer K Mägi R Krjutškov K Pettai K Whole-exome sequencing identifies a polymorphism in the BMP5 gene associated with SSRI treatment response in major depression. J Psychopharmacol (Oxford, England) (2013). 27(10):915–20. 10.1177/0269881113499829 23926243

[111] Uhr M Tontsch A Namendorf C Ripke S Lucae S Ising M Polymorphisms in the Drug Transporter Gene ABCB1 Predict Antidepressant Treatment Response in Depression. Neuron (2008). 57(2):203–9. 10.1016/j.neuron.2007.11.017 18215618

[112] Rasmussen ST Andersen JT Nielsen TK Cejvanovic V Petersen KM Henriksen T Simvastatin and oxidative stress in humans: A randomized, Double-blinded, Placebo-controlled clinical trial. Redox Biol (2016). 9:32–8. 10.1016/j.redox.2016.05.007 PMC4906137 27281490

[113] Wood SN Generalized Additive Models: An Introduction with R. 2nd ed. Taylor and Francis Inc. (2017). 10.1201/9781315370279.

[114] Fisher PM Ozenne B Svarer C Adamsen D Lehel S Baaré WF BDNF val66met association with serotonin transporter binding in healthy humans. Transl Psychiatry (2017). 7(2):e1029. 10.1038/tp.2016.295 28195567 PMC5438027

[115] Hastie TT The Elements of Statistical Learning Second Edition. Math Intell (2017). 7:228–30. 10.1007/b94608_7

[116] Polley EC van der Laan MJ Super Learner In Prediction. U.C. Berkeley Division of Biostatistics Working Paper Series . (2010). Working Paper 266.

[117] Rush AJ Fava M Wisniewski SR Lavori PW Trivedi MH Sackeim HA Sequenced treatment alternatives to relieve depression (STAR*D): Rationale and design. Control Clin Trials (2004). 25(1):119–42. 10.1016/S0197-2456(03)00112-0 15061154

