# Supplementary material for: Predicting Treatment Outcome in Major Depressive Disorder Using Serotonin 4 Receptor PET Brain Imaging, Functional MRI, Cognitive-, EEG-Based, and Peripheral Biomarkers: A NeuroPharm Open Label Clinical Trial Protocol
Source: Front Psychiatry. 2020 Jul 23;11:641. doi: 10.3389/fpsyt.2020.00641 (PMC7391965; doi:10.3389/fpsyt.2020.00641)
Supplement: Supplementary file 1 [file DataSheet_1.pdf]

Appendix 1

We here detail modality specific primary a priori hypotheses. Secondary hypotheses will be stated in the papers corresponding to each modality. Primary and secondary outcome measures are stated at clinicaltrials.gov. We will also perform replication analyses informed by existing literature and related clinical studies (e.g. CANBIND, iSPOT-D, EMBARC (Lam et al. 2016; Trivedi et al. 2016; Williams et al. 2011)).

Primary hypotheses

PET

- Patients with MDD differ in cerebral [^{11}C]SB207145 binding at baseline compared to healthy controls.
- [^{11}C]SB207145 binding at baseline in patients with MDD predicts remission after 8 weeks of pharmacological serotonergic intervention.
- After 8 weeks of serotonergic intervention, patients with remitter status have a significantly greater reduction in cerebral [^{11}C]SB207145 binding than non-responders.

MRI

- Change in dorsal attention network connectivity (dorsomedial prefrontal cortex) and the default mode network connectivity (anterior cingulate cortex and posterior cingulate cortex) are associated with serotonergic treatment response after 8 weeks.
- Lower amygdala reactivity to an emotional faces-paradigm at baseline predicts better serotonergic treatment response.
- Hippocampal volume at baseline predict treatment response.

EEG & ERP

- Patients with remitter status have greater alpha asymmetry (greater right than left hemisphere), higher theta band activity at anterior cingulate cortex (ACC), unstable vigilance regulation, and higher LDAEP at baseline.

Cognition

- Cognitive performance at baseline on affective cognitive tasks (Verbal Affective Memory Task 26, Emotional Recognition Task (eyes version)), social cognitive tasks (Moral Emotion task, Social Information Preference task) and cold cognitive tasks (Verbal Affective Memory Task 26, Letter-Number Sequence Task, Simple Reaction Time task) predict treatment response (remitter vs non-responder status) at week 8 and changes in clinical symptoms severity (changes in $HDRS_6$ scores from baseline to week 8 and 12).
- Patient performance on affective cognitive, social cognitive and cold cognitive tasks will improve after 12 weeks of serotonergic intervention.

Blood, urine and saliva biomarkers

- Systemic inflammation is associated with lower cognitive function at baseline and with treatment response after 8 weeks of serotonergic intervention.
- Systemic markers of oxidative stress are associated with response to serotonergic intervention.
- Restoration of HPA-axis dynamics in terms of the cortisol awakening response at week 8 is associated with treatment response to serotonergic intervention.

References

Lam, Raymond W., Roumen Milev, Susan Rotzinger, Ana C. Andreazza, Pierre Blier, Colleen Brenner, Zafiris J. Daskalakis, et al. 2016. "Discovering Biomarkers for Antidepressant Response: Protocol from the Canadian Biomarker Integration Network in Depression (CAN-BIND) and Clinical Characteristics of the First Patient Cohort." *BMC*

Psychiatry. https://doi.org/10.1186/s12888-016-0785-x.

Trivedi, Madhukar H., Patrick J. McGrath, Maurizio Fava, Ramin V. Parsey, Benji T. Kurian, Mary L. Phillips, Maria A. Oquendo, et al. 2016. "Establishing Moderators and Biosignatures of Antidepressant Response in Clinical Care (EMBARC): Rationale and Design." *Journal of Psychiatric Research*. https://doi.org/10.1016/j.jpsychires.2016.03.001.

Williams, Leanne M., A. J. Rush, Stephen H. Koslow, Stephen R. Wisniewski, Nicholas J. Cooper, Charles B. Nemeroff, Alan F. Schatzberg, and Evian Gordon. 2011. "International Study to Predict Optimized Treatment for Depression (ISPOT-D), a Randomized Clinical Trial: Rationale and Protocol." *Trials*. https://doi.org/10.1186/1745-6215-12-4.
